# Effectiveness of needle and syringe Programmes in people who inject drugs – An overview of systematic reviews

**DOI:** 10.1186/s12889-017-4210-2

**Published:** 2017-04-11

**Authors:** Ricardo M Fernandes, Maria Cary, Gonçalo Duarte, Gonçalo Jesus, Joana Alarcão, Carla Torre, Suzete Costa, João Costa, António Vaz Carneiro

**Affiliations:** 1 Center for Evidence-Based Medicine, Faculty of Medicine, University of Lisbonl, Av. Prof. Egas Moniz, 1649-028 Lisbon, Portugal; 2 Centre for Health Evaluation & Research (CEFAR), National Association of Pharmacies, Rua Marechal Saldanha, n°1, 1249-069 Lisbon, Portugal; 3 Portuguese Collaborating Centre of the IberoAmerican Cochrane Network-Cochrane Portugal Faculty of Medicine, University of Lisbon, Av. Prof. Egas Moniz, 1649-028 Lisbon, Portugal

**Keywords:** Needle and syringe programmes, Harm reduction interventions, People who inject drugs, HIV/Aids-Hcv-Hbv

## Abstract

**Background:**

Needle and syringe programmes (NSP) are a critical component of harm reduction interventions among people who inject drugs (PWID). Our primary objective was to summarize the evidence on the effectiveness of NSP for PWID in reducing blood-borne infection transmission and injecting risk behaviours (IRB).

**Methods:**

We conducted an overview of systematic reviews that included PWID (excluding prisons and consumption rooms), addressed community-based NSP, and provided estimates of the effect regarding incidence/prevalence of Human Immunodeficiency Virus (HIV), Hepatitis C virus (HCV), Hepatitis B virus (HBV) and bacteremia/sepsis, and/or measures of IRB. Systematic literature searches were undertaken on relevant databases, including EMBASE, MEDLINE, and PsychINFO (up to May 2015). For each review we identified relevant studies and extracted data on methods, and findings, including risk of bias and quality of evidence assessed by review authors. We evaluated the risk of bias of each systematic review using the ROBIS tool. We categorized reviews by reported outcomes and use of meta-analysis; no additional statistical analysis was performed.

**Results:**

We included thirteen systematic reviews with 133 relevant unique studies published between 1989 and 2012. Reported outcomes related to HIV (*n* = 9), HCV (*n* = 8) and IRB (*n* = 6). Methods used varied at all levels of design and conduct, with four reviews performing meta-analysis. Only two reviews were considered to have low risk of bias using the ROBIS tool, and most included studies were evaluated as having low methodological quality by review authors. We found that NSP was effective in reducing HIV transmission and IRB among PWID, while there were mixed results regarding a reduction of HCV infection. Full harm reduction interventions provided at structural level and in multi-component programmes, as well as high level of coverage, were more beneficial.

**Conclusions:**

The heterogeneity and the overall low quality of evidence highlights the need for future community-level studies of adequate design to support these results.

**Trial registration:**

The protocol of this systematic review was registered in Prospective Register of Systematic Reviews (PROSPERO 2015:CRD42015026145).

**Electronic supplementary material:**

The online version of this article (doi:10.1186/s12889-017-4210-2) contains supplementary material, which is available to authorized users.

## Background

People who inject drugs (PWID) experience high levels of morbidity and mortality. Drug-related harms include overdose, drug-related deaths, and blood-borne infections such as Human Immunodeficiency Virus (HIV), Hepatitis C (HCV), Hepatitis B (HBV), and bacteremia/sepsis. HCV is currently the most prevalent infectious disease affecting PWID, while HIV prevalence rates are lower. In an estimated total of 12.7 to 16 million PWID worldwide, it is believed that 1.2 million are infected with HIV [1] and 10 million are infected with HCV [2]. Sharing needles and syringes, as well as other injecting paraphernalia, is a key route of transmission of these infections [3].

Needle and syringe programmes (NSP) are thought to be a critical component of harm reduction interventions among PWID [3, 4]. The first NSP was established in the 1980s [5], in response to the global HIV epidemic, with the goal of providing access and encouraging the use of sterile injection paraphernalia by PWID. Since then, provision of these services has grown rapidly. Importantly, a shift in paradigm has favoured NSP as components of harm reduction or harm minimization policies, which focus on reducing all drug-related harms, i.e. preventing HIV, HBV and HCV infection, minimizing needle and syringe sharing and reuse, reducing the volume of discarded needles and syringes in the environment, and facilitating access to sterile paraphernalia. Furthermore, NSP may also promote the use of condoms and provide opportunistic relevant health information and services [3].

NSP are complex health interventions with several interacting components, such as behavioural changes in PWID and providers, a complex operating framework (users, providers, setting, health systems), and some degree of flexibility of interventions [6]. There is considerable variability among regions and countries in service provision, coverage and range of harm reduction interventions offered by NSP. In particular, a variety of measures have been developed to improve access to and use of sterile injecting equipment and to increase users’ choice. These include several methods of distribution or sale such as conventional NSP in fixed-sites, pharmacy-based distribution, dispensing machines and outreach programmes – often using a mobile van or bus, or through home-visits [3]. Generally, pharmacy and specialist needle exchange provides a wide range of harm reduction information and advice, along with clean needles and syringes and possibly injecting paraphernalia. The legal framework in which NSP operate also varies at country level [7]. Pharmacy-based NSP are known to be in place in at least Australia [8], Belgium, France, Ireland, Kyrgyzstan, the Netherlands, Portugal, Spain, Slovenia, Ukraine [7, 9], UK [10], and New Zealand [11].

Many systematic reviews on the effectiveness of interventions providing injecting equipment have been conducted to date. Overviews of reviews which compile information from these multiple systematic reviews, have also been published. The aim of these overviews is to provide end-users with a comprehensive and critical summary of the available evidence on this intervention. Overviews are of particular interest in this field because existing systematic reviews have focused on disparate questions, regarding either specific populations (e.g. country-specific evidence), specific types of interventions (e.g. different types of NSP provision) or, most commonly, specific outcomes (e.g. HIV or HCV transmission) [12–14]. Furthermore, there is variability in methods used in these systematic reviews, including the assessment of possible biases and the use of quantitative synthesis (meta-analysis). Nevertheless, to the best of our knowledge, the published overviews in this field have focused only on specific outcomes, i.e. transmission of HIV and/or HCV.

### Objectives

Our primary objective was to conduct an overview of systematic reviews that evaluated the evidence of the effectiveness of NSP for PWID across a range of different relevant outcomes, i.e. blood-borne infection transmission and injecting risk behaviours (IRB).

Our secondary objective was to assess how different aspects of NSP provision, including provider, setting, coverage and any related component delivered in parallel, such as harm reduction services and opiate substitution therapy, modified the effect of NSP, with a particular focus on pharmacy-driven NSP.

## Methods

We followed current guidance on the conduct of overviews of reviews, including recommendations from the Cochrane Collaboration [15] and guidance on public health intervention reviews by the Centre for Reviews and Dissemination of the University of York [16]. We also followed the recommendations from the PRISMA-P statement regarding reporting items that we considered applicable to this overview [17]. The protocol for this overview was registered at PROSPERO (CRD42015026145) available at http://www.crd.york.ac.uk/PROSPERO/display_record.asp?ID=CRD42015026145.

### Eligibility criteria

For inclusion in this overview, studies had to meet the following criteria:

1. Study design: systematic reviews, operationally defined as studies reporting a clearly stated set of objectives, eligibility criteria, a systematic search using two or more sources, a systematic presentation of the characteristics and findings of the included studies, and estimates of the size and direction of the effect of interventions presented as numerical data, on an individual study-level basis and/or with quantitative synthesis (meta-analysis);
2. Participants: PWID, defined as people who inject some form of drug at the beginning of the study. We excluded studies focusing exclusively on participants whose consumption was confined to prisons and consumption rooms, since these are populations with distinct characteristics from our target population;
3. Interventions: systematic reviews had to evaluate community-based NSP, defined as the supply of at least needles and syringes, with or without other injecting paraphernalia for the preparation and consumption of drugs;
4. Outcomes: required reported outcomes included the incidence and/or prevalence of blood-borne infections (HIV, HCV, HBV and bacteremia/sepsis), and/or measures of IRB (including but not limited to syringe re-use, borrowing, sharing, renting and lending).

When more than one review included exactly the same studies, the review that reported the most complete presentation of results was selected for inclusion in the overview.

### Search strategy and screening

Searches were undertaken on MEDLINE®In-Process & Other Non-Indexed Citations (from 1946 to 12th of May 2015), EMBASE (from 1974 to 12th of May 2015) and PsycINFO (from 1806 to 12th of May 2015) via the OVID SP interface. In addition, the following databases were searched: the NHS Economic Evaluation Database (NHS EED), the Database of Abstracts of Reviews of Effects (DARE), the Cochrane Database of Systematic Reviews (CDSR), the National Institute for Health and Clinical Excellence (NICE), the Campbell Library of Systematic Reviews and the Database of Promoting Health Effectiveness Reviews (DoPHER). The search strategies for each database are shown in a supplementary file (Additional file 1). No language or other types of restrictions were applied. In addition to the database searches, handsearching of the references of the included reviews was undertaken to identify further relevant studies.

Titles and abstracts of articles identified were screened independently by two authors (MC, JA), and classified as include, unclear or exclude. The full reports of all articles that classified as include or unclear were then obtained, and two authors (MC, GD) examined compliance of reviews with eligibility criteria, with a third author acting as an arbiter (RF).

### Data extraction

Data from reports of all included systematic reviews were extracted by two authors (MC, GD) and validated by a third author (RF), using a data extraction form designed and pre-piloted for this overview. The following general characteristics were extracted from each systematic review: publication details; study objectives; eligibility criteria (population, intervention, comparators, outcomes, study designs); any reported protocol; and methods used for search, screening, data extraction and synthesis. For each systematic review, we listed all included studies and evaluated whether they matched the eligibility criteria for this overview regarding population, interventions and outcomes. We then extracted the following results from the eligible group of studies, whenever reported at the systematic review level: study design; countries involved; characteristics of included participants (demographics, prevalence of HIV/HCV); description of interventions and any co-intervention, duration of intervention and follow-up; effect estimates from meta-analysis (if available) or at individual study-level, for each relevant outcome; and any subgroup or sensitivity analyses. The authors’ conclusions for each relevant outcome were also collected. We contacted the authors of reviews for relevant missing data.

### Assessment of methodological quality

At a study level, we extracted data on any risk of bias assessments of primary studies when performed and reported by reviewers in each systematic review, including tools used and summarized results. We also collected data on any reported evaluations of the quality of evidence concerning our outcomes of interest in included reviews, particularly those using the Grading of Recommendations Assessment, Development and Evaluation (GRADE) tool [18], as well as data on assessments of publication bias.

At a systematic review level, we assessed the methodological quality of each included systematic review using the Risk Of Bias In Systematic Reviews (ROBIS) tool [19]. Two review authors (MC, RF) performed quality assessments independently, using piloted decision rules. Disagreements regarding overall assessments were resolved through discussion, with a third reviewer serving as the final arbitrator (AVC). The rationale behind assessments was documented. We calculated measures of agreement and reliability between raters for each ROBIS domain.

### Data synthesis

We stratified the included systematic reviews by: (i) type of outcomes assessed (blood-borne infections, IRB), and (ii) type of analysis (with or without meta-analysis). We summarized data from the included reviews both in text and in summary tables and figures. When meta-analysis was performed, we report pooled estimates using the models and measures of effect reported by systematic review authors, with 95% confidence intervals (95% CI); we did not perform any additional statistical analysis. When reported, the accompanying *I* ^2^ values, which describe the percentage of total variation across studies that is due to heterogeneity rather than chance, were collected [20].

## Results

**Fig. 1:**
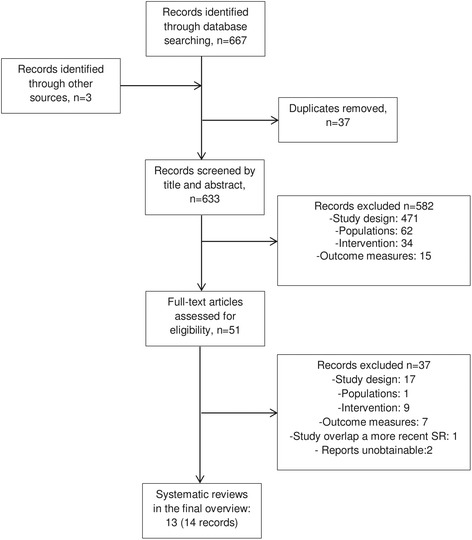
Screening decisions up to date as of 30Jun2015. Original search date 12May2015

The search and screening process is summarised in Fig. 1. A total of 667 citations were identified through the various database searches. Three additional records were identified in the reference lists of screened studies. After 37 duplicates were removed, we obtained 633 citations, which were screened by title and abstract. We excluded 582 citations as they did not meet the inclusion criteria, and the remaining 51 were screened full text. Thirty-five citations were further excluded, and two reports [21, 22] were unobtainable. Fourteen reports were thus included, corresponding to 13 systematic reviews, as two records referred to the same study [12, 23]. Within those publications three were reports from National Institutes/Expert Commitees in the US [24], UK [12] and Canada [25], while 10 were regular papers published in scientific journals.

### Description of included reviews

**Fig. 2:**
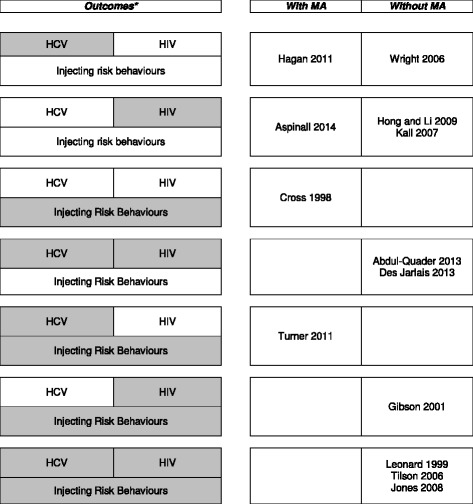
Studies selected for inclusion classified by outcome(s) reported and strategy for data synthesis. *Grey shaded boxes represent the outcome reported in the reviews indicated in the right column. “HIV”, ”HCV” and “Injecting risk behaviours” is used to classify the reviews that reported the impact of NSP in the number of HIV infections, number of HCV infections and change in injection behavior. The reviews are also presented in the right column by strategy used for data synthesis

Table 1 lists the key characteristics of the 13 included systematic reviews that evaluated the evidence of the effectiveness of NSP for PWID in the community setting. We classified reviews by type of outcomes reported and type of data analysis (Fig. 2).

**Table 1:** Key characteristics of the included systematic reviews and Risk of bias of each review

Authors, date of publication^a^	PICOS^b^	Dates of last search	Databases searched (*n*)	Risk of Bias and quality of evidence tools	Total number of studies/Number of studies relevant to the overview (designs)^c,d^	Risk of bias of the review (assessed using ROBIS tool)
Quantitative synthesis- meta-analysis						
Aspinall, 2014 [27]	P: PWID I: NSP (Specialist sites, pharmacies or outreach services) C: No, rare, or less exposure to NSP O: HIV incidence S: any	2012	CINAHL, Cochrane Library, EMBASE, IBSS, MEDLINE and PsycINFO (6)	Newcastle-Ottawa each study & GRADE to assess quality of evidence as a whole	12/12 (11 CC/Co; 1 OTH)	Low
Hagan, 2011 [34]	P: individuals who could conceivably have acquired HCV infection via administration of an illegal drug (PWID among them) I: interventions that could prevent HCV infection C:NR O: HCV prevalence or incidence rates, and measures of association with prevalence or incidence S: any	2010	Current Contents, Dissertation Abstracts, ERIC, NIH CRISP, MEDLINE, PsycINFO, Sociological Abstracts (7)	Developed their own quality scale	26/7 (7 CC/Co)	High
Turner, 2011 [32]	P: PWID in the UK I: NSP or OST (or combination) C: OST vs non OST; high vs low coverage of NSP O: HCV infection and IRB S: any	NR	PubMed and Web of Science (2)	None	4/3 (1 CC/Co; 2 OTH)	High
Cross, 1998 [26]	P: PWID I: NSP or educational programmes C: NR O: IRB S: experimental or quasi-experimental design	1995	AIDSLINE, MEDLINE, PsycINFO, Social Science Citation Index (4)	None	26/10 (1 RCT; 9 OTH)	High
Qualitative synthesis						
Abdul-Quader, 2013 [29]	P: PWID I: Structural interventions that address external factors to reduce HIV and HCV C: any quantitative comparison O: Changes in HIV/HCV in relation to intervention S: controlled or before & after	2011	Cochrane Central Register of Controlled Trials, EMBASE, LILACS, PsycINFO, PubMed, and the Web of Science/Web of Social Science (6)	WHO-Johns Hopkins 9-Point Rigour Scale.	15/15 (1 CC/Co; 14 OTH)	Unclear
Des Jarlais, 2013 [28]	P: PWID in low-and middle income and transitional countries (LMICs) I: NSP C: any O: changes in prevalence or incidence of HIV, HCV, HIV/ HCV co-infection or changes over time in newly reported HIV or HCV cases of infection S: any	2011	EMBASE, NLM Gateway, PubMed, Web of Science (4)	None	13/13 (13 OTH)	High
Hong and Li, 2009 [31]	P: People with HIV/ AIDS in China I: Any behavioural intervention C: Any O: Rate of needle-sharing S: Any	2008	AIDSLine, EBSCO, FirstSearch, PsycINFO, PubMed (4)	None	25/3 (1 RCT; 1 CC/Co; 1 OTH)	High
Jones, 2008 [12]	P: PWID I: NSP C: any O: IRB, HIV, HVC S: RCT, controlled non-randomised studies, controlled and uncontrolled before and after studies, cross-sectional studies, cohort studies, case-control studies and ecological studies	2008	ACP Journal Club, ASSIA, CINAHL, Cochrane Library, EMBASE, EPPI-Centre databases, Health Information Management Consortium, IBSS, MEDLINE, National Research Register Archive, OpenSIGLE, Project CORK, PsycINFO, Sociological Abstracts, SOMED (15)	Quality assessment tools developed by NICE Centre for Public Health Excellence, England and Wales and the Effective Public Health Practice Project, Canada	24/15 (2 RCT; 1 CC/Co; 12 OTH)	Low
Kall, 2007 [30]	P: PWID I: NSPs C: control or comparison groups O: HIV incidence or prevalence S: any	2005	MEDLINE and PsyclNFO (2)	None	16/16 (8 CC/Co; 8 OTH)	High
Wright, 2006 [33]	P: PWID I: primary prevention intervention targeting injecting drug use C: any O: HCV prevalence and incidence S: intervention or observational studies	2003	Cochrane Library, CINAHL, EMBASE, MEDLINE and PsycINFO (4)	Developed their own quality scale	18/12 (9 CC/Co; 3 OTH)	High
Tilson, 2006 [24]	P: PWID I: NSP C: With and without OST; Placebo. O: IRB, HCV and HIV S: NR	2006	Cochrane Library, Grey Literature Report, EMBASE, PubMed, PsycINFO, Social Science Abstracts, Web of Science, WorldCat (8)	GRADE	45/43 (28 CC/Co; 15 OTH)	Unclear
Gibson, 2001 [35]	P:PWID I:NSP C: any O:HIV, HBV, HCV and IRB S:any	1999	Medline and PsycINFO (2)	None	42/32 (19 CC/Co; 13 OTH)	High
Leonard, 1999 [25]	P: PWID I: NSP C: Any comparator group (but none was not allowed) O: HIV, HCV and IRB S: NR	1999	AIDSLINE, CINAHL, Cochrane Library, EMBASE, MEDLINE, PHEffect (6)	Quality assessment tools developed by the Effective Public Health Practice Project team, Canada	21/19 (15 CC/Co; 4 OTH)	Unclear

^a^Categorized by type of synthesis and listed by publication date

^b^PICOS: acronym for Population, Intervention, Comparator, Outcome and Study Design

^c^Classified by authors of the review

^d^CC/Co: Case-Control/Cohort; RCT: Randomized Control Trial; OTH: other (time-series designs, before-after studies, ecological studies)

The number of databases searched per review varied between two and 15, with the three most common sources being MEDLINE, EMBASE and PsycINFO. The dates of last search varied between 1995 [26] and 2012 [27]. Of 287 studies included in these 13 reviews, 200 studies matched the eligibility criteria for this overview, corresponding to 133 unique studies (a matrix with included studies by review is available in a supplementary file: Additional file 2). We included the complete set of studies from four reviews, as in the remaining reviews at least one study did not fulfill our eligibility criteria [27–30]. The number of relevant studies included per review ranged from three [31, 32] to 43 [24]. While there was some overlap in the included studies between reviews, the majority (68%) was included in one review only.

We found substantial variability in the criteria used by review authors to define and classify study designs, as well as types of designs of included studies. Only four reviews specified the types of study design in their eligibility criteria [12, 26, 29, 33]. Three reviews included randomized controlled trials (four RCTs overall) [12, 26, 31], while the remaining reviews included cohorts and case-controls (101 overall), as well as other study designs (time series, before/after studies or ecological studies) (95 overall).

Three of the included reviews were restricted to studies conducted in specific countries or economic regions (China, UK, and low- and middle-income countries) [28, 31, 32]. Nine reviews reported data on HIV, eight on HCV, and six on IRB. Seven reviews evaluated more than one of these outcomes. Four reviews used meta-analysis [26, 27, 32, 34], one of which using individual-participant data [32]. The remaining nine reviews used narrative synthesis [12, 24, 25, 28–31, 33, 35]. The reasons reported by the authors for conducting or not conducting meta-analysis varied between reviews, even when there was a degree of overlap between included studies.

### Methodological quality

Seven reviews reported having assessed the methodological quality of included studies [12, 24, 25, 27, 29, 33, 34]. In two reviews the authors developed ad hoc tools [33, 34], while the remaining reviews used previously existing instruments, often with modifications (tools used: Newcastle-Ottawa, GRADE, WHO-Johns Hopkins 9-Point Rigour Scale, Quality assessment tools developed by NICE Centre for Public Health Excellence, England and Wales and the Effective Public Health Practice Project, Canada) [12, 24, 25, 27, 29]. Only two instruments were used more than once, and all other instruments varied. Not all reviews reported the results of these assessments; when reported, most studies were considered to have low methodological quality, given methodological weaknesses and biases related to observational study designs. Only one quantitative review [27] incorporated quality in data synthesis, by conducting a sensitivity analysis. In reviews without meta-analysis, methodological quality and risk of bias was presented descriptively in the interpretation of study results. Two reviews [24, 27] reported using the GRADE tool to assess the quality of the body of evidence. One considered the overall quality of evidence as low [27], while the other, using an adaptation of GRADE, evaluated the quality of evidence as modest to moderate for different outcomes [24].

Using the ROBIS tool, only two of the included reviews were considered to have low risk of bias, whereas the remaining were considered to have either high (*n* = 8) or unclear risk of bias (*n* = 3) (summary of results available in a supplementary file: Additional file 3). The majority of reviews were rated as having high or unclear risk of bias across all ROBIS domains, i.e. study eligibility criteria, identification and selection of studies, data collection and study appraisal, synthesis and findings. Percentage of agreement between ROBIS raters varied between 77% and 92%, and weighted kappa (quadratic) between 0.82 and 0.95.

The results from the PRISMA-P statement applied to this overview are presented at Additional file 4.﻿

### Effects of NSP

#### HIV prevalence and/or incidence

Of nine reviews that reported data on HIV outcomes, two were at low risk of bias [12, 27], and only one performed meta-analysis [27]. The proportion of studies included in only one of these nine reviews was 71%.

##### Reviews with meta-analysis

The most recent review by Aspinall et al. included twelve studies (10 cohort, one case-control and one cross-sectional study), and was evaluated as having low risk of bias. The review reported a 34% “risk” reduction of HIV transmission (pooled effect estimate of 0.66; 95% CI: 0.43 to 1.01; *n* = 10 studies; I^2^ = 76%) in individuals exposed to NSP, compared with those who were not, or were less frequently, exposed to NSP. This estimate was obtained by pooling both adjusted and unadjusted effect sizes regardless of study design, in a random effects model, and pooling all types of effect measures (odds ratio, risk ratio, hazard ratio). Sensitivity analyses supported these results, with a statistically significant reduction in HIV transmission associated with NSP exposure in both higher quality studies (0.42; 95% CI: 0.22 to 0.81), and studies reporting relative risks or hazard ratios (0.60; 95% CI: 0.37 to 0.97). Subgroup analyses showed that studies using sequential follow-up (i.e. groups exposed to NSP and groups not exposed followed sequentially, and not concurrently) and studies recruiting post-1990 had lower effect estimates (0.21 [95% CI: 0.11 to 0.41] and 0.52 [95% CI: 0.28 to 0.95], respectively). Further, studies comparing 100% NSP coverage (i.e. clean needle and syringe used for 100% of injections) with <100% NSP coverage generated a pooled effect estimate of 0.58 (95% CI: 0.22 to 1.57). Quality of evidence was considered low using GRADE, and most of these results had substantial heterogeneity.

##### Reviews without meta-analysis

The quantitative results presented above are supported by most reviews with qualitative summaries of the evidence only, all of which published between 1999 and 2013. Differences between reviews could be found regarding eligibility criteria (e.g. type of NSP, population- vs individual-level outcomes, study design), search and screening methods, quality assessments and interpretation of study results. In most of these reviews, the authors described and contextualized results from each individual study or group of studies regarding HIV outcomes. Some reviews used vote counting to compare the number of positive studies with reduction in HIV transmission, with the number of negative studies. In the following paragraphs, we present the main results of these reviews, starting with those focusing on the impact of different aspects of NSP provision followed by results of reviews focusing exclusively on the comparison between NSP andno NSP.

Regarding aspects of NSP provision, Jones and colleagues reported results from a recent systematic review classified as low risk of bias, that focused on level of coverage, syringe dispensation policies, type of NSP, provision of additional harm-reduction services and of opiate substitution therapy [12]. Authors judged that the range of study designs, intervention approaches examined and outcomes precluded the use of meta-analysis. Only three studies focused on HIV outcomes. A low-quality study showed no significant trend for HIV prevalence when comparing primary sources of needles (pharmacies, fixed site NSP and van-based NSP), although HIV prevalence was lower among pharmacy users than in participants who reported using van or fixed site NSP (16% vs. 21% and 25%, respectively, *p* = 0.16) [36]. Another study [37] included in this review examined the impact of dispensation policies, and noted a decrease in HIV prevalence (based on testing or self-report) between the period of legal pharmacy syringe purchase and when up to five needles could be exchanged at newly established NSP (35% to 22%; *p* < 0.05). Finally, one moderate quality cohort study [38] also included in the review by Jones and colleagues evaluated different levels of harm reduction and found that a full harm reduction strategy (combination of methadone treatment and full participation in NSP) reduced the incidence of HIV when compared to incomplete or no harm reduction (incidence rate ratio: 0.32; 95% CI: 0.17 to 0.62).

In line with these results, a review with an overall unclear risk of bias by Abdul-Quader et al. [29] identified studies with structural-level NSP, ie interventions in which changes in policy and legal environment have facilitated an increased availability of sterile syringes, and focused on population-level outcomes. The operational definition of structural-level NSP was a minimum 50% coverage of PWID and distribution of 10 or more needles/syringe per PWID per year. Nine studies included in this review reported decreases in HIV prevalence, and three reported decreases in HIV incidence. All studies were non-randomized before-after comparisons or interrupted time series analyses, and most showed evidence of potential selection biases. The authors concluded that these results support NSP as a structural-level intervention to reduce population-level infection.

Four older published reviews with qualitative synthesis and an overall unclear or high risk of bias reported mixed findings regarding the impact of NSP on HIV incidence/prevalence. These reviews provide a historical perspective on the evolution of NSP implementation and evaluation. The first published review by Leonard et al. [25], was an update of a previous systematic review. Gibson et al. [35] included six studies published up to 1999 with a range of study designs, participants and settings, reporting on HIV outcomes. The Institute of Medicine’s evidence report [24], identified 12 relevant studies, including cohort, case-control and ecological designs, as well as studies using mathematical models. Finally, the review by Kall et al. [30] included 16 studies published up to 2005, only two of which were not included in other reviews from this overview.

All these reviews included landmark prospective cohort studies conducted in Montreal and Vancouver in the 1990s [39, 40], which found an association between NSP participation and higher risk of HIV seroconversion. This led Leonard et al. to conclude that there was methodologically weak evidence that NSP were not as effective as previously found in modifying HIV prevalence and incidence among PWID. Kall et al. used vote counting and reported that on most studies assessing seroincidence the effect of NSP was not significant, while four studies investigating seroprevalence at baseline were unfavourable to NSP. The authors also stated that in studies that found positive effects, confounders had not been adequately controlled for. However, based on epidemiological evidence that accumulated progressively, authors of the other reviews highlighted a number of selection biases that could account for these findings, including: the inclusion of high-risk cocaine injectors, who injected more often than heroin users; the limited number of needles and syringes that users could have access to in early NSP; and the ready availability of clean injecting equipment through pharmacies which could have attracted marginalized, particularly high-risk individuals.

Follow-up studies in the same settings, conducted after expanding and adjusting NSP (e.g. by allowing unlimited distribution of needles/syringes, increasing the number of access points, and offering different distribution methods), found no such increase in risk, or a decrease in HIV prevalence. Gibson et al. [35] used vote counting and considered there was substantial evidence that NSP were effective in preventing HIV seroconversion. Tilson et al. [24] included four ecological studies that found an association between HIV prevention programmes that include NSP with reduced prevalence of HIV in urban settings. Based on the weakness of these studies designs, this evidence was considered modest using a modified GRADE approach. Further, moderate evidence was found that multi-component HIV prevention programmes that include NSP reduce intermediate HIV risk behaviour.

The Institute of Medicine report highlighted how almost all published studies originate in North America, Western Europe, and Australia [24]. Two additional reviews were restricted to specific populations by geographical or economical source. Hong and Li summarized evidence from two studies conducted solely in China [31], while Des Jarlais et al. focused on 13 studies of 11 NSPs with high-coverage conducted in low/middle-income countries [28]. In both cases, results from included studies generally supported the effectiveness of NSP in reducing HIV. Des Jarlais et al. reported a reduction of HIV prevalence in four studies (from −3% to −15%), of estimated HIV incidence in three studies (from −11/100 to −16/100 person-years at risk), and of newly reported nationwide cases in three national reports (from −30% to −93.3%). Conversely, increases in HIV prevalence were found in two studies (from +5.6% to +15.8%) and one national report (+37.6%) included in the review by Des Jarlais et al. The authors considered that, if high coverage is achieved, NSP appear to be as effective in low/middle-income as in high-income countries [28].

#### HCV prevalence and/or incidence

Eight included reviews synthesized the evidence on the use of NSP in preventing HCV prevention in PWID. One was rated as being at low [12], three at unclear [24, 25, 29] and four at high risk of bias [28, 32–34], including two reviews that used meta-analysis [32, 34]. The proportion of studies included exclusively in one of these reviews was 88%. Both reviews with quantitative and qualitative synthesis showed mixed results.

##### Reviews with meta-analysis

Two reviews [32, 34], both published in 2011 and rated as being at high risk of bias, used meta-analysis.

Hagan et al. [34] included 7 studies focusing on NSP and HCV outcomes (6 cohort and 1 case-control study), all from North America. The pooled analysis of all studies, using random effects models and with all measures of effect converted to relative risks, showed an increase in the risk of HCV acquisition with NSP (relative risk, 1.62; 95% CI: 1.04 to 2.52). There was considerable heterogeneity (I^2^ = 81%), but no subgroup or sensitivity analyses were performed, and study quality was not explicitly reported or considered in the analysis. Authors cautioned against possible volunteer bias in studies which found an association between HCV acquisition and stand-alone NSP participation, given that exchange programmes attract and retain higher-risk PWID.

The second review by Turner et al. performed individual participant-data meta-analysis based on studies solely conducted in the UK [32]. High NSP coverage (100% versus <100% needles per injection) reduced the risk of new HCV infection (adjusted odds ratio 0.48; 95% CI: 0.25 to 0.93) (*n* = 833 participants, from 3 studies). Full harm reduction (on opiate substitution treatment plus high NSP coverage) further reduced the odds of new HCV infection (adjusted odds ratio 0.21; 95% CI: 0.08 to 0.52). Results were adjusted for gender, homelessness, crack injection and duration of injection.

##### Reviews without meta-analysis

Earlier reviews with qualitative synthesis included many studies which were not included in the previously mentioned reviews with meta-analysis. These reviews reported mixed results mostly summarized using vote counting, and we found distinct interpretations of findings. We first present the main results of reviews that focused on the influence of different aspects of NSP provision, and then results of reviews focusing exclusively on the comparison of NSP versus no NSP intervention.

The low risk of bias review by Jones et al. [12] focused on the impact of different aspects of NSP provision and included two studies with HCV outcomes. Both of these studies also included HIV outcomes, and results were consistent for both infections: no difference in HCV prevalence was found when comparing primary sources of needles (pharmacies, fixed site NSP and van-based NSP), and a full harm reduction intervention (including NSP with opiate replacement therapy) was found to reduce the incidence of HCV when compared to no harm reduction (incidence rate of 3.5/100 person-years vs. 23.2/100 person-years with an incidence rate ratio of 0.15; 95% CI: 0.06 to 0.40) [38].

The most recent review by Abdul-Quader et al. [29]*,* classified as having an unclear risk of bias, included six structural intervention studies with before-after design and outcome data on HCV biomarkers published up to 2011, all of which reported a reduction in HCV prevalence after NSP implementation. It should be noted, as described above, that these studies were restricted to NSP with high-coverage based on the number of syringes distributed and the number of PWID in the local population, and that only studies with pharmacy sales in conjunction with NSP were included (and not programmes exclusively focused on pharmacy sale or distribution).

While mostly focused on HIV and IRB outcomes, the Institute of Medicine’s report from 2006 [24] also considered the impact of NSP on HCV prevention. Five studies provided moderate evidence (adjusted GRADE) that NSP had significantly less impact on transmission and acquisition of HCV than on HIV, which was attributed by study authors to the apparent failure of these programmes in providing other injecting equipment. There was however one case-control study that showed a considerable decrease in HCV acquisition. Another of these earlier reviews, by Wright et al. [33] based on a World Health Organization review [41], included 12 relevant studies. Authors highlighted how included observational studies from Europe and Australia showed statistically significant reductions in anti-HCV prevalence or incidence in the early 1990s (shortly after the introduction of NSP), but the trend did not continue in the following years. Also, reported negative studies conducted in the US failed to identify an association between NSP and HCV incidence. On the other hand, two studies with expanded harm reduction included in this review, demonstrated a statistically significant reduction in HCV. Authors of this review concluded that a more effective response to HCV prevention in drug users should include provision of new interventions, such as behavioural interventions and distribution of other injecting paraphernalia alongside needle injection material.

The earliest review, by Leonard and colleagues [25], focused on three studies which, similarly to HIV outcomes, provided conflicting and methodologically weak evidence that NSP were not associated with decreases in the risk of new infections with HCV (one earlier study showing reduced transmission, and two later studies failing to show any association).

Regarding low/middle-income and transitional-economy countries, Jarlais and colleagues [28] reviewed four relevant studies, and found a decrease in HCV prevalence (range − 4.2% to −10.2%) in three of those studies, while HCV incidence remained stable in the fourth study. The authors considered that, as with HIV, if high coverage was achieved, NSP appeared to be as effective as in high-income countries.

#### Injecting risk behaviours

Six reviews reported data on IRB outcomes. One was at low risk of bias [12], two were at unclear [24, 25] and three at high risk of bias [26, 32, 35]. Two reviews performed meta-analysis [26, 32]. Specific IRB outcomes reported by each review and study varied. The proportion of studies included in only one of these six reviews was 77%.

##### Reviews with meta-analysis

The systematic review by Turner et al. [32], considered to be at high risk of bias, included an individual participant-data meta-analysis reporting UK study data on self-reported IRB outcomes of needle sharing and mean number of injections in the last month. Full harm reduction strategy was associated with a reduced risk in both of these outcomes, with an adjusted odds ratio of 0.52 (95% CI: 0.32 to 0.83) for the former, and an adjusted mean difference of −20.8 (95% CI: -27.3 to −14.4) for the latter (both *n* = 2143 participants).

An earlier review by Cross et al. [26], classified as having high risk of bias, pooled data from 10 studies (one RCT and nine observational studies) published up to 1995 across a range of IRB outcomes, including sharing and lending syringes, condom use, injecting frequency and bleach use. NSP were effective in reducing high risk drug use and sexual behaviours (weighted group mean 0.28; 95% CI, 0.21 to 0.35) (*n* = 1675). The magnitude of the effect size was affected by outcome and study design: the largest group effect of NSP was to reduce sharing, followed by lending and injecting, and results from pre-post-test designs showed larger effects than the randomized study.

##### Reviews without meta-analysis

The review by Jones et al. provided relevant study results on the possible influence of different aspects of NSP provision on IRB outcomes [12]. Two poor quality studies included in this review found that higher syringe coverage and participation in opiate substitution therapy alongside NSP reduced injection risk behaviours among drug users. Further, one cohort study and one cross-sectional study suggested that PWID who obtained their needles exclusively from NSP were less likely to engage in high risk behaviours than those who obtained them via secondary distribution, and in turn the latter had less IRB than those who obtained no needles directly or indirectly from NSP. On the other hand, evidence from two RCTs also included in Jones et al. review suggested that NSP setting does not impact on injection risk behaviours. One of these trials compared pharmacy sales only with NSP exchange plus pharmacy sales [42], and the other compared hospital and community-based NSP [43]. As supplementary evidence, three poor quality cross-sectional studies found that mobile van sites and vending machines may attract younger PWID with higher risk profiles. Finally, evidence from three cross-sectional studies, also included in this review, suggested that syringe dispensation policies had a limited impact on behavioural outcomes such as sharing, but some impact on syringe re-use.

Reviews with narrative summaries provided largely similar results regarding the overall effect of NSP. Tilson et al. included 18 cohorts in their systematic review that examined the impact of NSP on drug-related risks [24]. Thirteen studies found a reduction in self-reported needle sharing. One study found injection frequency to decrease, and four other studies showed no difference in this outcome. Authors cautioned that nearly all programmes combined needle and syringe exchange with other components such as outreach, risk reduction education, condom distribution, bleach distribution, education on needle disinfection, and referrals to substance abuse treatment and other health and social services. Evidence was judged to be moderate (adjusted GRADE). The authors reviewed the evidence for pharmacy sales and physician-based prescriptions and concluded that pharmacy dispensation, free of criminal penalty, was an alternative strategy to make sterile needles and syringes available to PWID.

Two earlier reviews [25, 35], already described in the previous sections of the results, also analysed IRB outcomes. Gibson et al. identified 23 studies published up to 1999, and using vote counting concluded that there was substantial evidence that NSP are effective in preventing IRB. Leonard et al. reviewed 19 low quality primary studies and evidence supported an effect of NSP in modifying risk related injection practices.

## Discussion

### Summary of main results

In this overview of systematic reviews examining the effectiveness of NSP for PWID in reducing blood-borne infection transmission and injecting risk behaviours, we identified 13 systematic reviews contributing with 133 unique studies, which were mostly observational. Methods used in these reviews varied at all levels of review design and conduct. Only two reviews were considered to have low risk of bias by reviewers [12, 27] and most included studies were evaluated as having low methodological quality. The quality of evidence, when assessed, was considered low or modest to moderate. Nine reviews reported outcome data on HIV prevalence/incidence, eight on HCV, and six on IRB. Meta-analysis was performed in four of these reviews.

Our interpretation of the findings is that the overall results of the included systematic reviews are supportive of the effectiveness of NSP in reducing HIV transmission and IRB among PWID, as well as in reducing HCV infection, although the latter to lesser extent. The overall quality of the evidence is higher for HIV transmission and IRB than for HCV infection. However, for HCV infection, the strength of the evidence increases (because studies’ results are more consistent) if the intervention under consideration is not solely NSP, but includes other components such as opiate replacement treatment, in a strategy of full harm reduction intervention.

Furthermore it is well known that sharing other injecting equipment (e.g. swabs, cookers, water and filters) is an important route of transmission of blood-borne infections, particularly in the case of HCV. In addition, clean injecting equipment is also available through sources other than NSP (dilution bias) [44, 45]. This overview did not identify any studies evaluating the effects of paraphernalia distribution at reducing the incidence or prevalence of HCV. One further aspect is that individual NSP intervention studies are prone to selection (volunteer) bias, as these exchange programmes attract and retain higher-risk PWID. Taken together, these aspects may have contributed to some mixed results reported in the systematic reviews and individual studies addressing HCV infection.

To sum up, aspects of NSP provision may be relevant, including structural-level NSP (i.e. high-level coverage), and multi-component programmes including full harm reduction seem to benefit all outcomes more than individual NSP.

### Overall completeness and applicability of evidence

In this field of public health, interventions and results are highly influenced by economic, legal, ethical, social and cultural circumstances. In the past, results from some of the included studies led to major political decisions and fuelled controversial debates, particularly in the US. There are considerable differences in perspectives regarding the role and provision of harm reduction services, which impact study design and conduct and selection of participants, and thus affect the validity and generalizability of study results. This may at least partially explain why we found heterogeneity in study results and time trends, although there was consistency in many outcomes. Further, while the majority of the published research presented in different reviews originated from North America and Europe, one of the reviews [28] supported part of these results for both high and low/middle-income countries. Importantly, provision of NSP interventions is low at a global level, particularly in countries where the incidence and prevalence of both HIV and HCV is highest.

Aspects of NSP provision are also key to decision-making, and few included reviews examined how different types of NSP provision impact on effectiveness. NSP are extremely diverse in their design, staffing, characteristics of participants, operation and programme delivery policies. Further, PWID are diverse populations with different preferences, behaviours, and life circumstances, who often have difficulty in accessing formal healthcare services. As such, the potential impact of NSP in reducing injecting related-harms is limited by the extent to which the programmes provide effective access to sterile injecting equipment and other services, and are therefore able to attract their potential clients [46, 47].

Results from this overview support the importance of high-level coverage and comprehensive services provided by many NSP in reducing bloodborne infections and risk behaviours. We should note that outcomes which were not included in this review may be relevant to evaluate possible benefits of NSP. For example, pharmacy access to sterile needles and syringes has been found to provide specific benefits in addition to those available through specialist NSPs [12]. Importantly, the coexistence of different modes of injecting equipment delivery, as well as tailoring services offered at different venues, addresses several barriers encountered by PWID, as modalities for improving syringe availability are likely complementary and not competitive [48–50].

The additional benefits provided through this complementary role of pharmacies may have prompted Governments of Australia, Ireland, Spain (Basque Country) and UK to remunerate pharmacies for the important public health role provided through NSP [8, 51–55]. As pharmacy-based NSP gradually expand, it is important to pursue future research with a focus on this setting.

### Quality of the evidence

The results hereby presented have to be treated with considerable caution. The methodological quality of the included systematic reviews varied, but a majority of reviews were considered to be at high risk of bias. Importantly, we found considerable heterogeneity and inadequacies in methods used by different reviews, at all stages of the design, conduct and reporting of the review. Differences between eligibility criteria and search and screening methods likely explain a striking mismatch between included studies, despite some reviews having similar research questions and covering relatively close publication periods. Assessment of quality of included studies was seldom performed, with the use of different tools for assessment of risk of bias, some of which were developed ad hoc with no validation, and hardly any use of grading of quality of evidence. We also found variability in the decision as to whether or not to perform quantitative synthesis, even when the body of evidence was similar. Few review authors chose to present pooled estimates from meta-analyses, and when present, there was variation in models, choice of outcome measures and use of sensitivity and subgroup analyses. Other authors opted to present narrative summaries of results, often with vote counting, but there were discrepancies in the interpretation of trial results. Thus, there were obstacles in summarizing, comparing and synthesizing results from all included reviews.

These factors also reflect the challenges that review authors had in summarizing results from individual studies, most of which were judged to be of low methodological quality. Classification of study design was discrepant between reviews, but most studies were observational, at either individual or population-level. These designs are limited in establishing causality, and we found different approaches regarding adjustment for possible mediators and confounders. Further, a range of selection biases were identified and explored in some landmark studies, including volunteer bias of highest risk users into NSP. Less biased evaluations of NSP would require the use of randomization, for example at a cluster level, but there are many ethical, scientific and practical challenges. Other aspects to consider are the challenges in selecting, comparing and pooling different outcome measures and measurement tools when assessing HIV and HCV infection, as well as needle and syringe sharing and injecting frequency. A final limitation of this body of evidence is that studies generally were not designed to allow for separate examination of different aspects of NSP provision. As most public health interventions, NSP are complex health interventions with several interacting components, multilevel factors (users, providers, setting, health systems), and some degree of flexibility of interventions allowed in the real-world setting. Hence, challenges will likely persist in identifying the independent contribution of improving access to sterile needles and syringes, both at review and individual study level.

### Agreements and disagreements with other reviews and potential biases

Two other overviews of reviews were published on this topic [12, 14]. The first overview, published in 2008 [12], was undertaken as a section of an extensive review of the effectiveness and cost-effectiveness of NSPs for PWID to inform guidance on the optimal provision of NSPs from the perspective of the UK National Health Service. The authors concluded that none of the included systematic reviews examined HCV in any depth and that there was insufficient evidence to support the effectiveness of NPS on HCV incidence/prevalence. On the other hand, there was tentative and sufficient evidence to support the effectiveness of NSP on HIV incidence/prevalence and on self-reported IRB, respectively. The second overview was an updated overview published in 2014 [13, 14] and focused on the effectiveness of different harm reduction interventions, including NSP, in preventing IRB, HIV and HCV transmission among PWID [14]. This review was related to guidance from the European Centre for Disease Prevention and Control and the European Monitoring Centre for Drugs and Drug Addiction. Taking into consideration the individual conclusions of the core included systematic reviews, as well as the number of individual studies showing positive findings (i.e. a reduction of IRB, HIV and/or HCV incidence/prevalence), the authors concluded that there was: 1) sufficient review-level evidence of effectiveness in relation to IRB; 2) tentative review-level evidence to support the effectiveness of NSP in reducing HIV transmission among PWID; and 3) insufficient review-level evidence to support the effectiveness of NSP in reducing HCV transmission.

The field of overviews is relatively new, and this type of study design has been gaining momentum as a valuable knowledge synthesis methodology that can collate extensive information to facilitate the uptake and application of knowledge by decision-makers [56]. However, published overviews show considerable variation in their methods and reporting due to the unique methodological challenges inherent in summarizing and synthesizing evidence from different heterogeneous sources [57, 58]. Reviews in public health pose additional challenges, as methods are likely to vary more, with less quantitative analysis and different approaches to synthesizing and interpreting evidence.

Previous overviews have used different approaches to review eligibility, selection, quality assessment and analysis. Also, there were differences in the framework used to classify included reviews (e.g. core/supplementary), the type of summary and synthesis used (e.g. vote counting according to study or review results), and the conclusions and evidence statements. In comparison, the strengths of our overview include the use of strict eligibility criteria, the evaluation of methodological quality of included systematic reviews, and the categorization of review by type of analysis and outcome. We used the novel ROBIS tool [19], which was developed and validated to overcome limitations seen in previous instruments to assess the conduct and reporting of systematic reviews. This tool allowed us to stratify review quality using clear and transparent criteria, thus providing support to our confidence in each review’s findings. Further, by presenting pooled estimates of the effect of NSP in different outcomes taken from reviews that performed meta-analysis, we provide the opportunity for readers to quantify the direction and magnitude of this effect. We also highlight the need to use this quantitative data to assess the likely relevance of findings.

There are a number of limitations to our overview. While we identified and listed all unique studies included in each review and assessed the degree of overlap between reviews, we did not assess these studies individually, as other overviews have [57]. We focused on results from different reviews, which had some degree of overlap, and thus have a risk of double counting results, both for qualitative and quantitative reviews. Further, the application of ROBIS was challenging, although measures of agreement between raters were relatively adequate, likely due to the use of piloted decision rules.

## Conclusions

The findings of this overview of systematic reviews allow the conclusion that there is moderate quality evidence that NSP is likely effective in reducing HIV transmission and IRB among PWID, and that there is low to moderate quality evidence that NSP in the context of a comprehensive harm reduction strategy is likely effective in reducing HCV infection. Full harm reduction interventions provided at structural level and in multi-component programmes seem to be more beneficial. The scarcity and the lack of robust quality of evidence highlights the need for future community-level studies of adequate design to support these conclusions, as well as to address the impact of different aspects of NSP provision. Future reviews and possibly overviews of reviews should use standardized methods and frameworks to improve comparability, synthesis and interpretation of findings.

## Additional files

Additional file 1: Search Strategies. (DOCX 19 kb)

Additional file 2: List of primary studies included in each review. (DOCX 26 kb)

Additional file 3: Summary results from ROBIS evaluation performed by two assessors (MC&RF). (DOCX 13 kb)

Additional file 4: Prisma Checklist. (DOCX 14 kb)

## Abbreviations

CC: Case-Control study
CDSR: Cochrane Database of Systematic Reviews
CEFAR: Centre for Health Evaluation & Research
CH: Cohort study
DARE: Database of Abstracts of Reviews of Effects
DoPHER: Campbell Library of Systematic Reviews and the Database of Promoting Health Effectiveness Reviews
GRADE: Grading of Recommendations Assessment, Development and Evaluation
HBV: Hepatitis B virus
HCV: Hepatitis C virus
HIV: Human Immunodeficiency Virus
IRB: injecting risk behaviours
NHS EED: National Health System Economic Evaluation Database
NICE: National Institute for Health and Clinical Excellence
NSP: Needle and syringe programmes
OTH: other study design (time-series designs, before-after studies, ecological studies)
PRISMA: Preferred Reporting Items for Systematic Reviews and Meta-Analyses
PWID: people who inject drugs
RCT: Randomized Control Trial

## References

[1] UNAIDS (2014). UNAIDS: the gap report.

[2] WHO (2007). Guidance on prevention of viral hepatitis B and c among people who inject drugs.

[3] Torre C (2009). Syringe exchange Programmes in the context of harm reduction. Arq Med.

[4] Ball AL, Rana S, Dehne KL (1998). HIV prevention among injecting drug users: responses in developing and transitional countries. Public Health Rep.

[5] van den Hoek JA, van Haastrecht HJ, Coutinho RA (1989). Risk reduction among intravenous drug users in Amsterdam under the influence of AIDS. Am J Public Health.

[6] Craig P, Dieppe P, Macintyre S, Michie S, Nazareth I, Petticrew M (2008). Developing and evaluating complex interventions: new guidance.

[7] European Monitoring Centre for Drugs and Drug Addiction (EMCDDA) (2015). European drug report: data and statistics.

[8] The Pharmacy Guild of Australia: Community pharmacy roadmap program development template. Needle and syringe program. 2010; Barton: The Pharmacy Guild of Australia.

[9] Gay Men’s Health Crisis (2009). Syringe exchange programs around the world: global context.

[10] National Institute for Health and Care Excellence (NICE) (2014). Needle and syringe Programmes, NICE public health guidance.

[11] Sheridan J, Henderson C, Greenhill N, Smith A (2005). Pharmacy-based needle exchange in New Zealand: a review of services. Harm Reduct J.

[12] Jones L, Pickering L, Sumnall H, Mcveigh J, Mark A, Bellis M. A review of the effectiveness and cost-effectiveness of needle and syringe Programmes for injecting drug users. 2008, Liverpool: Centre for Public Health, Liverpool John Moores University.

[13] Palmateer N, Kimber J, Hickman M, Hutchinson S, Rhodes T, Goldberg D (2010). Evidence for the effectiveness of sterile injecting equipment provision in preventing hepatitis C and human immunodeficiency virus transmission among injecting drug users: a review of reviews. Addiction.

[14] MacArthur GJ, van Velzen E, Palmateer N, Kimber J, Pharris A, Hope V, Taylor A, Roy K, Aspinall E, Goldberg D, Rhodes T, Hedrich D, Salminen M, Hickman M, Hutchinson SJ (2014). Interventions to prevent HIV and hepatitis C in people who inject drugs: a review of reviews to assess evidence of effectiveness. Int J Drug Policy.

[15] Higgins JPT, Green S (editors). Cochrane Handbook for Systematic Reviews of Interventions Version 5.1.0 [updated March 2011]. The Cochrane Collaboration, 2011. Available from http://handbook.cochrane.org/.

[16] Centre for Reviews and Dissemination (2008). Systematic reviews: CRD’s guidance for undertaking reviews in health care.

[17] Moher D, Shamseer L, Clarke M, Ghersi D, Liberati A, Petticrew M (2015). Preferred reporting items for systematic review and meta-analysis protocols (PRISMA-P) 2015 statement. Syst Rev.

[18] Guyatt GH, Oxman AD, Vist GE, Kunz R, Falck-Ytter Y, Alonso-Coello P (2008). GRADE: an emerging consensus on rating quality of evidence and strength of recommendations. BMJ.

[19] Whiting P, Savović J, Higgins JPT, Caldwell DM, Reeves BC, Shea B (2016). ROBIS: a new tool to assess risk of bias in systematic reviews was developed. J Clin Epidemiol.

[20] Higgins JPT, Thompson SG, Deeks JJ, Altman DG (2003). Measuring inconsistency in meta-analyses. BMJ.

[21] Takacs I, Demetrovics Z (2009). The efficacy of needle exchange programs in the prevention of HIV and hepatitis infection among injecting drug users. Psychiatr Hung.

[22] De Lima M, Justo L, Formigoni M (2005). Needle exchange in Sao Paulo city: a harm reduction strategy for injection drug users. J Bras Psiquiatr.

[23] Jones L, Pickering L, Sumnall H, McVeigh J, Bellis MA (2010). Optimal provision of needle and syringe programmes for injecting drug users: a systematic review. Int J Drug Policy.

[24] Tilson H, Aramrattana A, Bozzette S, Celentano D, Falco M, Hammett T (2006). Preventing HIV Infecrtion among injecting drug users in high risk countries: an assessment of the evidence.

[25] Leonard L, Forrester L, Navarro C, Hansen J, Doucet C (1999). The effectiveness of needle exchange programs in modifying HIV-related outcomes: a systematic review of the evidence 1997–1999.

[26] Cross J, Saunders CM, Bartelli D (1998). The effectiveness of educational and needle exchange programs: a meta-analysis of HIV prevention strategies for injecting drug users. Qual Quant.

[27] Aspinall E, Nambiar D, Goldberg D, Hickman M, Weir A, Van Velzen E (2014). Are needle and syringe programmes associated with a reduction in hiv transmission among people who inject drugs: a systematic review and meta-analysis. Int J Epidemiol.

[28] Des Jarlais DC, Feelemyer JP, Modi SN, Abdul-Quader A, Hagan H (2013). High coverage needle/syringe programs for people who inject drugs in low and middle income countries: a systematic review. BMC Public Health.

[29] Abdul-Quader AS, Feelemyer J, Modi S, Stein ES, Briceno A, Semaan S, Horvath T (2013). Effectiveness of structural-level needle/syringe programs to reduce HCV and HIV infection among people who inject drugs: a systematic review. AIDS Behav.

[30] Kall K, Hermansson U, Amundsen EJ, Ronnback K, Ronnberg S. The Effectiveness of Needle Exchange Programmes for HIV Prevention A Critical Review. J Glob Drug Policy Pract. 2007;1(3). http://www.globaldrugpolicy.org/Issues/Vol%201%20Issue%203/The%20Effectiveness%20of%20Needle%20Exchange.pdf. Accessed 20 Aug 2015.

[31] Hong Y, Li X (2009). HIV/AIDS behavioral interventions in China: a literature review and recommendation for future research. AIDS Behav.

[32] Turner KME, Hutchinson S, Vickerman P, Hope V, Craine N, Palmateer N (2011). The impact of needle and syringe provision and opiate substitution therapy on the incidence of hepatitis C virus in injecting drug users: pooling of UK evidence. Addiction.

[33] Wright NMJ, Tompkins CNE (2006). A review of the evidence for the effectiveness of primary prevention interventions for hepatitis C among injecting drug users. Harm Reduct J.

[34] Hagan H, Pouget ER, Des Jarlais DC (2011). A systematic review and meta-analysis of interventions to prevent hepatitis C virus infection in people who inject drugs. J Infect Dis.

[35] Gibson DR, Flynn NM, Perales D (2001). Effectiveness of syringe exchange programs in reducing HIV risk behavior and HIV seroconversion among injecting drug users. AIDS.

[36] Miller CL, Tyndall M, Spittal P, Li K, Palepu A, Schechter MT (2002). Risk-taking behaviors among injecting drug users who obtain syringes from pharmacies, fixed sites, and mobile van needle exchanges. J Urban Health.

[37] Singer M, Himmelgreen D, Weeks MR, Radda KE, Martinez R (1997). Changing the environment of AIDS risk: findings on syringe exchange and pharmacy sales of syringes in Hartford, CTs. Med Anthropol.

[38] Van Den Berg C, Smit C, Van Brussel G, Coutinho R, Prins M (2007). Full participation in harm reduction programmes is associated with decreased risk for human immunodeficiency virus and hepatitis C virus: evidence from the Amsterdam cohort studies among drug users. Addiction.

[39] Bruneau J, Lamothe F, Franco E, Lachance N, Désy M, Soto J (1997). High rates of HIV infection among injection drug users participating in needle exchange programs in Montreal: results of a cohort study. Am J Epidemiol.

[40] Strathdee S, Patrick D, Currie S, Cornelisse P, Rekart M, Montaner J (1997). Needle exchange is not enough: lessons from the Vancouver injecting drug use study. AIDS.

[41] Wright N, Millson C (2005). What is the evidence for the effectiveness of interventions to reduce hepatitis C infection and the associated morbidity.

[42] Fisher DG, Fenaughty AM, Cagle HH, Wells RS (2003). Needle exchange and injection drug use frequency: a randomized clinical trial. J Acquir Immune Defic Syndr.

[43] Masson C, Sorensen J, Perlman D, Shopshire M, Delucchi K, Chen T (2007). Hospital- versus community-based syringe exchange: a randomized controlled trial. AIDS Educ Prev.

[44] Cox J, De P, Morissette C, Tremblay C, Stephenson R, Allard R (2008). Low perceived benefits and self-efficacy are associated with hepatitis C virus (HCV) infection-related risk among injection drug users. Soc Sci Med.

[45] Heinzerling KG, Kral AH, Flynn NM, Anderson RL, Scott A, Gilbert ML (2007). Human immunodeficiency virus and hepatitis C virus testing services at syringe exchange programs: availability and outcomes. J Subst Abus Treat.

[46] Kral AH (2003). What is it about needle and syringe programmes that make them effective for preventing HIV transmission?. Int J Drug Policy.

[47] Small W (2005). Examining barriers to syringe access among injection drug users. Int J Drug Policy.

[48] WHO (2008). Policy guidelines for collaborative TB and HIV Services for Injecting and Other Drug Users – an integrated approach.

[49] Coffin P (2000). Syringe availability as HIV prevention: a review of modalities. J Urban Health.

[50] Moatti JP, Vlahov D, Feroni I, Perrin V, Obadia Y (2001). Multiple access to sterile syringes for injection drug users: vending machines, needle exchange programs and legal pharmacy sales in Marseille, France. Eur Addict Res.

[51] European Monitoring Centre for Drugs and Drug Addiction (EMCDDA) (2016). Harm reduction overview for Ireland.

[52] Programa de Intercambio de Jeringuillas, País Vasco. http://www.observatoriocarteraservicios.com/iniciativas/programa-de-intercambio-de-jeringuillas-pais-vasco#. Accessed 29 Dec 2015.

[53] NHS England: Improving Health and Patient Care through Community Pharmacy - Evidence Resource Pack. 2013; London. https://www.england.nhs.uk/wp-content/uploads/2013/12/comm-pharm-res-pack.pdf. Accessed 29 Dec 2015.

[54] Community Pharmacy Contract - Enhanced Services. http://www.wales.nhs.uk/sites3/page.cfm?orgid=498&pid=7552. Accessed 29 Dec 2015.

[55] NHS Care Services. Local Services. http://www.communitypharmacyscotland.org.uk/nhs-care-services/services/local-services/. Accessed 29 Dec 2015.

[56] Hartling L, Vandermeer B, Fernandes RM (2014). Systematic reviews, overviews of reviews and comparative effectiveness reviews: a discussion of approaches to knowledge synthesis. Evid-Based Child Heal.

[57] Hartling L, Chisholm A, Thomson D, Dryden DM. A descriptive analysis of overviews of reviews published between 2000 and 2011. PLoS One. 2012;7:e49667. http://dx.doi.org/10.1371/journal.pone.0049667. 10.1371/journal.pone.0049667 PMC3499476 23166744

[58] Pieper D, Buechter R, Jerinic P, Eikermann M (2012). Overviews of reviews often have limited rigor: a systematic review. J Clin Epidemiol.

