# Supplementary material for: Effectiveness of needle and syringe Programmes in people who inject drugs – An overview of systematic reviews
Source: BMC Public Health. 2017 Apr 11;17:309. doi: 10.1186/s12889-017-4210-2 (PMC5387338; doi:10.1186/s12889-017-4210-2)
Supplement: Supplementary file 1 — Search Strategies. (DOCX 19 kb) [file 12889_2017_4210_MOESM1_ESM.docx]

**S1 Table. Search Strategies**

| **Database: Embase <1974 to 2015 May 12>, Ovid MEDLINE(R) In-Process & Other Non-Indexed Citations and Ovid MEDLINE(R) <1946 to Present>, PsycINFO <1806 to May Week 1 2015>** |
| --- |
| 1 Needle-Exchange Programs/  2 Harm Reduction/  3 Harm reduc$.tw.  4 Needles/sd [Supply & Distribution]  5 Syringes/sd [Supply & Distribution]  6 (NSP or NEP or NSEP or NSPs or NEPs or NSEPs or SEP or SEPs).tw.  7 ((needle$ or syringe$ or inject$ or paraphernalia or equipment) adj3 (exchang$ or supply$ or access$ or provision or provid$ or distribut$ or dispens$ or program$ or disposal or service$ or centre$ or scheme$ or center$ or site$1 or facilities or facility or scheme$ or area$ or pharmacy or pharmacies or unit$ or pack$1 or kit$1 or mobile van dispensing machine or vending machine or outreach program)).tw.  8 or/1-7  9 ((incidence or prevalence or low$ or reduc$ or prevent$ or decreas$) adj5 (HIV or hepatitis or HCV or HBV or blood-borne or blood borne or BBV or transmission or infection$ or virus$ or bacteria$ or viral or morbidity or mortality or death$ or overdose$ or seroconversion or seroprevalence)).tw.  10 exp HIV/  11 exp Hepatitis C/ or exp Hepatitis B/  12 Blood-Borne Pathogens/  13 HIV infections/  14 virus diseases/  15 Human Immunodeficiency Virus.tw.  16 HIV.tw.  17 HCV.tw.  18 hepatitis c.tw.  19 hepatitis B.tw.  20 HBV.tw.  21 AIDS/  22 exp Endocarditis, Bacterial/  23 exp Sepsis/  24 Sepsi*.tw.  25 Blood Poisoning*.tw.  26 Pyaemia*.tw.  27 Pyemia*.tw.  28 Endocarditis.tw.  29 Endocarditides.tw.  30 ((Sharing or re-use) adj2 (needle$ or syringe$ or inject$ or paraphernalia or equipment)).tw.  31 or/9-30  32 Substance Abuse, Intravenous/  33 Drug users/  34 ((substance$1 or drug$1 or stimulant$) adj3 (abuse or misuse or dependen$ or use$2 or usage or addict$ or inject$ or intravenous$)).tw.  35 (idu$ or Inject$ drug user$).tw.  36 (pwid or "people who inject drug$1").tw.  37 or/32-36  38 8 and 31 and 37  39 meta-analysis/  40 review literature/  41 meta-analy$.tw.  42 metaanal$.tw.  43 (systematic$ adj4 (review$ or overview$)).mp. [mp=ti, ab, sh, hw, tn, ot, dm, mf, dv, kw, nm, kf, px, rx, ui, tc, id, tm]  44 meta-analysis.pt.  45 review.pt.  46 review.ti.  47 review literature.pt.  48 39 or 40 or 41 or 42 or 43 or 44 or 45 or 46 or 47  49 case report/  50 letter.pt.  51 historical article.pt.  52 review of reported cases.pt.  53 review,multicase.pt.  54 49 or 50 or 51 or 52 or 53  55 48 not 54  56 38 and 55  57 remove duplicates from 56 |
| **Database: Database of promoting health effectiveness reviews (DoPHER)** |
| 1 Needle Exchange  2 Syringe Exchange  3 Supervised Injecting  4 1 or 2 or 3 |
| **Database: The Campbell Collaboration Library of systematic reviews** |
| (Supervised and Inject*) or (needle or syringe) and exchange |
| **Database: Nice evidence search databases: systematic reviews** |
| (needle exchange) or (syringe exchange) |
| **DARE and NHS EED** |
| Needle exchange or syringe exchange |
| **Cochrane Library** |
| (needle exchange) or (syringe exchange) |
