# Supplementary material for: Effectiveness of needle and syringe Programmes in people who inject drugs – An overview of systematic reviews
Source: BMC Public Health. 2017 Apr 11;17:309. doi: 10.1186/s12889-017-4210-2 (PMC5387338; doi:10.1186/s12889-017-4210-2)
Supplement: Supplementary file 2 — List of primary studies included in each review. (DOCX 26 kb) [file 12889_2017_4210_MOESM2_ESM.docx]

| **Reference** | [**Cross, 1998**](file:///C:\Users\maria.Cary\Dropbox\CEMBE_CEFAR\1_PTS\Overview%20Literature\extração%20dados\DEF%20v6%20data%20extracted%2021122015.xlsx#'DOPHER_5_Cross_1998 '!A1) | [**Leonard, 1999**](file:///C:\Users\maria.Cary\Dropbox\CEMBE_CEFAR\1_PTS\Overview%20Literature\extração%20dados\DEF%20v6%20data%20extracted%2021122015.xlsx#DOPHER_14_Leonard_1999!A1) | [**Gibson, 2001**](file:///C:\Users\maria.Cary\Dropbox\CEMBE_CEFAR\1_PTS\Overview%20Literature\extração%20dados\DEF%20v6%20data%20extracted%2021122015.xlsx#OEP_305_Gibson_2001!A1) | [**Wright, 2005**](file:///C:\Users\maria.Cary\Dropbox\CEMBE_CEFAR\1_PTS\Overview%20Literature\extração%20dados\DEF%20v6%20data%20extracted%2021122015.xlsx#OEP_195_Wright_2006!A1) | **Tilson, 2006** | **Kall 2007** | [**Hong and Li, 2009**](file:///C:\Users\maria.Cary\Dropbox\CEMBE_CEFAR\1_PTS\Overview%20Literature\extração%20dados\DEF%20v6%20data%20extracted%2021122015.xlsx#'Hong and Li 2009'!A1) | **Jones, 2008** | [**Turner, 2011**](file:///C:\Users\maria.Cary\Dropbox\CEMBE_CEFAR\1_PTS\Overview%20Literature\extração%20dados\DEF%20v6%20data%20extracted%2021122015.xlsx#OEP_63_Turner_2011!A1) | [**Hagan, 2011**](file:///C:\Users\maria.Cary\Dropbox\CEMBE_CEFAR\1_PTS\Overview%20Literature\extração%20dados\DEF%20v6%20data%20extracted%2021122015.xlsx#OEP_99_Hagan_2011!A1) | **Des** [**Jarlais, 2013**](file:///C:\Users\maria.Cary\Dropbox\CEMBE_CEFAR\1_PTS\Overview%20Literature\extração%20dados\DEF%20v6%20data%20extracted%2021122015.xlsx#OEP_68_Jarlais_2013!A1) | [**Abdul-Quader, 2013**](file:///C:\Users\maria.Cary\Dropbox\CEMBE_CEFAR\1_PTS\Overview%20Literature\extração%20dados\DEF%20v6%20data%20extracted%2021122015.xlsx#OEP_14_Abdul_2013!A1) | [**Aspinal, 2014**](file:///C:\Users\maria.Cary\Dropbox\CEMBE_CEFAR\1_PTS\Overview%20Literature\extração%20dados\DEF%20v6%20data%20extracted%2021122015.xlsx#OEP_37_Aspinall_2014!A1) |
| --- | --- | --- | --- | --- | --- | --- | --- | --- | --- | --- | --- | --- | --- |
| Donoghoe 1989 | x |  | x |  |  |  |  |  |  |  |  |  |  |
| Hartgers 1989 |  |  | x |  |  |  |  |  |  |  |  |  |  |
| Hart 1989 | x |  |  |  | x |  |  |  |  |  |  |  |  |
| van den Hoek 1989 | x |  |  |  | x |  |  |  |  |  |  |  |  |
| Wolk 1990 | x |  |  |  |  |  |  |  |  |  |  |  |  |
| Ljungberg 1991 | x |  |  |  |  | x |  |  |  |  |  |  |  |
| Klee 1991 | x |  | x |  | x |  |  |  |  |  |  |  |  |
| Scully 1991 | x |  |  |  |  |  |  |  |  |  |  |  |  |
| Donoghoe 1992 |  |  | x |  |  |  |  |  |  |  |  |  |  |
| Hartgers 1992 |  |  | x |  | x |  |  |  |  |  |  |  |  |
| van Ameijden 1992 |  |  | x |  | x | x |  |  |  |  |  |  |  |
| Keene 1993 | x |  | x |  | x |  |  |  |  |  |  |  |  |
| FrischerAnd Elliot 1993 |  |  | x |  |  |  |  |  |  |  |  |  |  |
| Longshore 1993 | x |  |  |  |  |  |  |  |  |  |  |  |  |
| VanAmeijden 1993 |  |  |  | x |  |  |  |  |  |  |  |  |  |
| Hagan 1993 | x |  |  |  | x |  |  |  |  |  |  |  |  |
| Frischer 1993 |  |  | x |  |  |  |  |  |  |  |  |  |  |
| Des Jarlais 1994 |  |  | x |  |  |  |  |  |  |  |  |  |  |
| vanAmeijden 1994 |  |  | x |  | x |  |  |  |  |  |  |  |  |
| Watters 1994 |  |  | x |  | x |  |  |  |  |  |  |  |  |
| Hagan 1994 |  |  | x |  |  |  |  |  |  |  |  |  |  |
| Oliver 1994 |  |  | x |  |  |  |  |  |  |  |  |  |  |
| Des Jarlais 1995 |  |  |  |  | x |  |  |  |  |  |  |  |  |
| Peak 1995 |  |  | x |  |  |  |  |  |  |  |  |  |  |
| Hagan 1995 |  | x | x | x | x |  |  |  |  | x |  |  |  |
| KleeAnd Morris 1995 |  |  | x |  |  |  |  |  |  |  |  |  |  |
| Guydish 1995 |  |  | x |  |  |  |  |  |  |  |  |  |  |
| Paone 1995 |  |  | x |  |  |  |  |  |  |  |  |  |  |
| van Ameijden, 1995 |  |  |  |  |  | x |  |  |  |  |  |  |  |
| Des Jarlais 1996 |  |  | x |  | x | x |  |  |  |  |  |  | x |
| Schoenbaum 1996 |  |  | x |  | x | x |  |  |  |  |  |  | x |
| van Haastrecht et al., 1996 |  |  |  |  | x |  |  |  |  |  |  |  |  |
| Bruneau 1997 |  | x | x |  | x | x |  |  |  |  |  |  | x |
| Singer 1997 |  | x | x |  |  |  |  | x |  |  |  |  |  |
| Lamother 1997 |  |  |  |  |  |  |  |  |  | x |  |  |  |
| Morrison 1997 |  | x |  |  |  |  |  |  |  |  |  |  |  |
| Paone 1997 |  | x |  |  |  |  |  |  |  |  |  |  |  |
| Hurley 1997 |  |  |  |  | x | x |  |  |  |  |  |  |  |
| Keene & Stimson 1997 |  | x |  |  |  |  |  |  |  |  |  |  |  |
| Patrick 1997 |  |  | x |  | x | x |  |  |  |  |  |  | x |
| Strathdee 1997 |  |  | x |  | x | x |  |  |  |  |  |  |  |
| van Ameijden And Coutinho 1997 |  |  | x |  | x |  |  |  |  |  |  |  |  |
| Vlahov 1997 |  | x | x |  | x |  |  |  |  |  |  |  |  |
| Kipke 1997 |  | x |  |  |  |  |  |  |  |  |  |  |  |
| Lurie & Drucker 1997 |  | x |  |  |  |  |  |  |  |  |  |  |  |
| Perlman 1997 |  | x |  |  |  |  |  |  |  |  |  |  |  |
| Remis 1998 |  | x |  |  |  |  |  |  |  |  |  |  |  |
| Archibald 1998 |  | x |  |  |  |  |  |  |  |  |  |  |  |
| Bluthenthal 1998 |  | x | x |  |  |  |  |  |  |  |  |  |  |
| Goldberg 1998 |  |  |  | x |  |  |  |  |  |  |  | x |  |
| Gray 1998 |  |  |  |  |  |  |  |  |  |  | x |  |  |
| Guydish 1998 |  |  | x |  |  |  |  |  |  |  |  |  |  |
| Heimer 1998 |  | x |  |  |  |  |  |  |  |  |  |  |  |
| MacGowan 1998 |  | x |  |  |  |  |  |  |  |  |  |  |  |
| Paone 1998 |  | x |  |  |  |  |  |  |  |  |  |  |  |
| Vogt 1998 |  | x |  |  |  |  |  |  |  |  |  |  |  |
| Obadia 1999 |  |  |  |  |  |  |  | x |  |  |  |  |  |
| Broadhead 1999 |  |  | x |  |  |  |  |  |  |  |  |  |  |
| Hagan 1999 |  | x | x | x |  |  |  |  |  |  |  |  |  |
| Paone 1999 |  | x |  |  |  |  |  |  |  |  |  |  |  |
| Smyth 1999 |  |  |  | x |  |  |  |  |  |  |  |  |  |
| Rockwell 1999 |  |  |  |  |  |  |  | x |  |  |  |  |  |
| Schechter 1999 |  |  | x |  | x | x |  |  |  |  |  |  | x |
| Khoshnood 2000 |  |  |  |  |  |  |  | x |  |  |  |  |  |
| Riley 2000 |  |  |  |  |  |  |  | x |  |  |  |  |  |
| Bluthenthal 2000 |  |  |  |  | x |  |  |  |  |  |  |  |  |
| Cox 2000 |  |  |  |  | x |  |  |  |  |  |  |  |  |
| Des Jarlais 2000 |  |  |  |  | x |  |  |  |  |  |  |  |  |
| Hagan 2000 |  |  |  |  | x |  |  |  |  |  |  |  |  |
| MacDonald 2000 |  |  |  | x |  |  |  |  |  |  |  |  |  |
| Somaini 2000 |  |  |  | x |  |  |  |  |  |  |  |  |  |
| Taylor 2000 |  |  |  | x |  |  |  |  |  |  |  |  |  |
| Mannson 2000 |  |  |  | x | x |  |  |  |  |  |  |  | x |
| Marmor 2000 |  |  |  |  | x |  |  |  |  |  |  |  |  |
| Monterroso 2000 |  |  |  |  | x | x |  |  |  |  |  |  | x |
| Vertefeuille 2000 |  |  |  |  | x |  |  |  |  |  |  |  |  |
| Longshore 2001 |  |  |  |  | x |  |  |  |  |  |  |  |  |
| Goldberg 2001 |  |  |  | x |  |  |  |  |  |  |  | x |  |
| Patrick 2001 |  |  |  | x |  |  |  |  |  | x |  |  |  |
| Valente 2001 |  |  |  |  |  | x |  |  |  |  |  |  | x |
| Gibson 2002 |  |  |  |  | x |  |  |  |  |  |  |  |  |
| HOI 2002 |  |  |  |  |  | x |  |  |  |  |  |  |  |
| Miller 2002 |  |  |  |  |  |  |  | x |  |  |  |  |  |
| Hutchinson 2002 |  |  |  | x |  |  |  |  |  |  |  |  |  |
| Thorpe 2002 |  |  |  |  |  |  |  |  |  | x |  |  |  |
| Safaeian et al., 2002 |  |  |  |  | x |  |  |  |  |  |  |  |  |
| Wood 2002 |  |  |  |  | x |  |  |  |  |  |  |  |  |
| Fisher 2003 |  |  |  |  |  |  |  | x |  |  |  |  |  |
| Amundsen 2003 |  |  |  |  | x | x |  |  |  |  |  |  |  |
| MacDonald 2003 |  |  |  |  | x | x |  |  |  |  |  |  |  |
| Caiaffa 2003 |  |  |  |  |  |  |  |  |  |  | x |  |  |
| Wood 2003 |  |  |  |  | x |  |  |  |  |  |  |  |  |
| Bluthenthal 2004 |  |  |  |  |  |  |  | x |  |  |  |  |  |
| Bruneau 2004 |  |  |  |  | x |  |  |  |  |  |  |  |  |
| Kral 2004 |  |  |  |  |  |  |  | x |  |  |  |  |  |
| Ouellet 2004 |  |  |  |  | x |  |  |  |  |  |  |  |  |
| Rhodes 2004 |  |  |  |  |  |  |  | x |  |  |  |  |  |
| Hagan 2004 |  |  |  |  |  |  |  |  |  | x |  |  |  |
| Lin 2004 |  |  |  |  |  |  | x |  |  |  |  |  |  |
| Schilling 2004 |  |  |  |  |  |  |  | x |  |  |  |  |  |
| Des Jarlais 2005b 2005 |  |  |  |  | x |  |  |  |  |  |  | x |  |
| Des Jarlais 2005 |  |  |  |  | x |  |  |  |  |  |  | x | x |
| Huo 2005 |  |  |  |  | x |  |  |  |  |  |  |  |  |
| Hope 2005 |  |  |  |  |  |  |  |  |  |  |  | x |  |
| Vazirian et al., 2005 |  |  |  |  | x |  |  |  |  |  |  |  |  |
| Hammett 2006 |  |  |  |  | x |  |  |  |  |  | x | x |  |
| Ramirez-Jonville 2006 |  |  |  |  |  |  |  |  |  |  |  | x |  |
| Des Jarlais 2007 |  |  |  |  |  |  | x |  |  |  | x | x |  |
| Masson 2007 |  |  |  |  |  |  |  | x |  |  |  |  |  |
| Millson 2007 |  |  |  |  |  | x |  | x |  |  |  |  |  |
| Roy 2007 |  |  |  |  |  |  |  |  |  | x |  |  |  |
| Smyth 2007 |  |  |  |  |  |  |  |  |  |  |  | x |  |
| Van Den Berg 2007 |  |  |  |  |  |  |  | x |  |  |  |  | x |
| Wu 2007 |  |  |  |  |  |  | x |  |  |  | x |  |  |
| Bluthenthal 2007 |  |  |  |  |  |  |  | x |  |  |  |  |  |
| CDC Iranian MOH 2007 |  |  |  |  |  |  |  |  |  |  | x |  |  |
| Azim 2008 |  |  |  |  |  |  |  |  |  |  | x |  |  |
| Azim 2009 |  |  |  |  |  |  |  |  |  |  | x |  |  |
| Craine 2009 |  |  |  |  |  |  |  |  | x |  |  |  |  |
| Des Jarlais 2009 |  |  |  |  |  |  |  |  |  |  |  | x |  |
| Holtzaman 2009 |  |  |  |  |  |  |  |  |  | x |  |  |  |
| Des Jarlais 2009b |  |  |  |  |  |  |  |  |  |  |  | x |  |
| University of the West of Scotland 2010 |  |  |  |  |  |  |  |  | x |  |  |  |  |
| Des Jarlais 2010 |  |  |  |  |  |  |  |  |  |  |  | x |  |
| Kerr 2010 |  |  |  |  |  |  |  |  |  |  |  | x | x |
| Austraudkirne 2010 |  |  |  |  |  |  |  |  |  |  | x |  |  |
| CDC 2010 |  |  |  |  |  |  |  |  |  |  | x |  |  |
| Hammett 2010 |  |  |  |  |  |  |  |  |  |  | x |  |  |
| Bruneau 2011 |  |  |  |  |  |  |  |  |  |  |  | x | x |
| Uuskula 2011 |  |  |  |  |  |  |  |  |  |  | x |  |  |
| Hope 2011 |  |  |  |  |  |  |  |  | x |  |  |  |  |
| Topp 2011 |  |  |  |  |  |  |  |  |  |  |  | x |  |
| Hammett 2012 |  |  |  |  |  |  |  |  |  |  | x |  |  |
