# Supplementary material for: Effectiveness of needle and syringe Programmes in people who inject drugs – An overview of systematic reviews
Source: BMC Public Health. 2017 Apr 11;17:309. doi: 10.1186/s12889-017-4210-2 (PMC5387338; doi:10.1186/s12889-017-4210-2)
Supplement: Supplementary file 3 — Summary results from ROBIS evaluation performed by two assessors (MC&RF). (DOCX 13 kb) [file 12889_2017_4210_MOESM3_ESM.docx]

| **Authors, date of publication^(1)^** | **1. Study eligibility criteria** | | **2. Identification and selection of studies** | | **3. Data collection and study appraisal** | | **4. Synthesis and findings** | | **RISK OF BIAS IN THE REVIEW** | |
| --- | --- | --- | --- | --- | --- | --- | --- | --- | --- | --- |
|  | **MC** | **RF** | **MC** | **RF** | **MC** | **RF** | **MC** | **RF** | **MC** | **RF** |
| Aspinall, 2014 | LOW | LOW | LOW | UNCLEAR | UNCLEAR | UNCLEAR | LOW | LOW | LOW | LOW |
| Des Jarlais, 2013 | LOW | LOW | UNCLEAR | UNCLEAR | HIGH | HIGH | HIGH | HIGH | HIGH | HIGH |
| Abdul-Quader, 2013 | LOW | LOW | LOW | LOW | UNCLEAR | LOW | LOW | UNCLEAR | UNCLEAR | UNCLEAR |
| Hagan, 2011 | UNCLEAR | UNCLEAR | LOW | LOW | UNCLEAR | UNCLEAR | HIGH | HIGH | HIGH | HIGH |
| Turner, 2011 | UNCLEAR | HIGH | HIGH | HIGH | HIGH | HIGH | HIGH | HIGH | HIGH | HIGH |
| Hong, 2009 | UNCLEAR | HIGH | HIGH | HIGH | HIGH | HIGH | HIGH | HIGH | HIGH | HIGH |
| Jones, 2008 | LOW | LOW | LOW | LOW | LOW | LOW | UNCLEAR | UNCLEAR | LOW | LOW |
| Kall, 2007 | HIGH | HIGH | HIGH | HIGH | HIGH | HIGH | HIGH | HIGH | HIGH | HIGH |
| Wright, 2006 | LOW | LOW | UNCLEAR | UNCLEAR | UNCLEAR | UNCLEAR | HIGH | HIGH | HIGH | HIGH |
| Tilson, 2006 | UNCLEAR | UNCLEAR | LOW | LOW | UNCLEAR | UNCLEAR | UNCLEAR | UNCLEAR | UNCLEAR | UNCLEAR |
| Gibson, 2001 | HIGH | HIGH | HIGH | HIGH | HIGH | UNCLEAR | HIGH | HIGH | HIGH | HIGH |
| Leonard, 1999 | UNCLEAR | UNCLEAR | UNCLEAR | UNCLEAR | LOW | LOW | UNCLEAR | UNCLEAR | UNCLEAR | UNCLEAR |
| Cross, 1998 | LOW | UNCLEAR | HIGH | HIGH | HIGH | HIGH | HIGH | HIGH | HIGH | HIGH |
|  |  |  |  |  |  |  |  |  |  |  |
| **% of agreement** | 77% | | 92% | | 85% | | 92% | | 100% | |

**S3 Table. Summary results from ROBIS evaluation performed by two assessors (MC&RF).**
